# Mitochondrial quality control beyond PINK1/Parkin

**DOI:** 10.18632/oncotarget.23799

**Published:** 2018-01-02

**Authors:** Sophia von Stockum, Elena Marchesan, Elena Ziviani

**Affiliations:** Department of Biology, University of Padova, Padova, Italy; Istituto IRCCS San Camillo, Lido di Venezia, Venezia, Italy

**Keywords:** mitochondria, mitophagy pathways, quality control, neurodegeneration, neuroscience

Neurons strictly rely on proper mitochondrial function and turnover. They possess a high energy requirement which is mostly fueled by mitochondrial oxidative phosphorylation. Moreover the unique morphology of neurons implies that mitochondria need to be transported along the axons to sites of high energy demand. Finally, due to the non-dividing state of neurons, cellular mitosis cannot dilute dysfunctional mitochondria, which can produce harmful by-products such as reactive oxygen species (ROS) and thus a functioning mechanism of quality control (QC) is essential. The critical impact of mitochondria on neuronal function and viability explains their involvement in several neurodegenerative diseases such as Parkinson’s disease (PD) [1].

**Figure 1:**
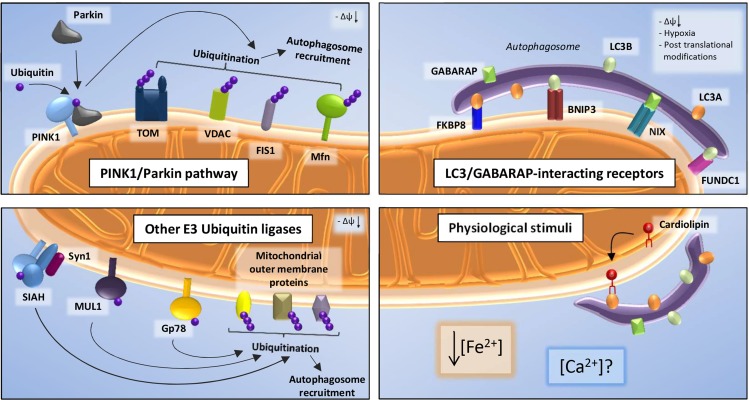
Upon loss of transmembrane potential, the kinase PINK1 accumulates on the OMM and recruits the E3 ubiquitin ligase Parkin Parkin-mediated ubiquitination on several mitochondrial proteins leads to the clearance of dysfunctional mitochondria (upper left). Following additional conditions (i.e. hypoxia, post translational modification), LC3-II and GABARAP, components of the autophagosomal membrane, can interact with OMM mitophagy receptors such as FKBP8, BNIP3, NIX and FUNDC1, thus inducing mitophagy (upper right). Mitochondrial recruitment of LC3 can also be mediated by Cardiolipin; moreover, calcium ([Ca^2+^]) and iron ([Fe^2+^]) levels could play a role in PINK1/Parkin-independent mitophagy (lower right). Finally, E3 Ubiquitin ligases other than Parkin, such as Gp78, MUL1 and SIAH1 contribute to ubiquitination of OMM target proteins (lower left).

Mitophagy, a selective form of autophagy, is employed by cells to degrade dysfunctional mitochondria in order to maintain a healthy mitochondrial network, a process also called mitochondrial QC. In order for mitophagy to take place it essentially requires molecules that on the one hand sense the dysfunctional mitochondria and on the other hand tag the latter for autophagic degradation. Most studies on mitophagy are focused on a canonical pathway including the PD-related proteins PINK1 and Parkin. PINK1 is a kinase that recruits the E3 ubiquitin ligase Parkin to depolarized mitochondria, where it ubiquitinates several target proteins on the outer mitochondrial membrane (OMM) leading to their proteasomal degradation and serving as a signal to recruit the autophagic machinery [1] (Figure 1, upper left corner). A large number of studies on PINK1/Parkin mitophagy are based on treatment of cell lines with the uncoupler CCCP collapsing the mitochondrial membrane potential (ΔΨm), as well as overexpression of Parkin, conditions that are far from physiological [1]. Furthermore, Parkin translocation to mitochondria in neuronal cells occurs only under certain stimuli and is much slower, possibly due to their metabolic state and low endogenous Parkin expression [2]. Thus, in recent years several studies have highlighted pathways of mitophagy induction that are independent of PINK1 and/or Parkin and could act in parallel or addition to the latter. We want to discuss what is known about these QC mechanisms and hypothesize on their role in neuronal physiology and neurodegeneration.

Alternative mitophagy pathways can basically branch into or parallel the PINK1/Parkin pathway at any point ranging from Parkin translocation to autophagosome formation. However, most studies are focused on two steps: ubiquitination of OMM proteins by E3 ubiquitin ligases other than Parkin, or involvement of OMM mitophagy receptors that target mitochondria to autophagosomes independently of Parkin-induced ubiquitination. These receptors directly interact with LC3-II and GABARAP, components of the autophagosomal membrane, through a specific binding motif, the LC3-interacting region (LIR). A few examples of these receptors are FKBP8, a member of the FK506-binding protein family, recruiting LC3A to damaged mitochondria in response to depolarization [3], BNIP3 and NIX, pro-apoptotic members of the Bcl2-family, and FUNDC1 which target mitochondria to autophagosomes interacting directly with LC3B and GABARAP in response to hypoxia or phosphorylation of the receptor itself [4] (Figure, upper right corner). Another non-protein molecule that can directly interact with LC3-II is the inner mitochondrial membrane (IMM) phospholipid Cardiolipin (CL), which contains a LIR motif. When CL is externalized from the IMM to the OMM in response to depolarization, it can recruit LC3, thus inducing mitophagy [5] (Figure, lower right corner).

Additionally, E3 Ubiquitin ligases other than Parkin, such as Gp78, MUL1 and SIAH1 have been reported to contribute to ubiquitination of OMM target proteins such as the mitochondrial fusion protein Mitofusin (Mfn). Gp78 ubiquitinates Mfn1 and Mfn2 inducing their proteasomal degradation, thereby regulating mitochondrial dynamics, mobility and mitophagy [6]. MUL1 was also shown to ubiquitinate Mfn in *Drosophila* and to regulate mitophagy [4]. SIAH1 acts in a complex together with PINK1 and syniphilin-1 that promotes mitophagy in the absence of Parkin. PINK1 can recruit synphilin-1 to the mitochondria, which in turn recruits SIAH1, subsequently ubiquitinating mitochondrial proteins, leading to LC3 recruitment (Figure, lower left corner). Interestingly, PINK1 PD-related mutants do not form this complex pointing at a possible involvement of this pathway in the disease [7].

How these different mitophagy pathways are regulated and differentially activated under physiological conditions and which role they play in neuronal function and neurodegeneration still needs investigation. Several studies have convincingly shown that elimination of dysfunctional mitochondria is taking place in brains and neurons as measured e.g. by co-localization of mitochondria with autophagosomes or by measurement of mitochondrial mass in the presence of lysosomal inhibitors [2]. As mentioned above, PINK1 and Parkin certainly play a role in this but might not be the only actors. It is still unclear which physiological stimuli trigger mitophagy induction *in vivo*. Recently, an iron chelator was identified as an inducer of PINK1/Parkin-independent mitophagy without collapsing ΔΨm [8] hinting to iron depletion as a novel physiological stimulus, although the proper mechanism needs investigation. This could be particularly relevant in PD and other neurodegenerative diseases, since impaired iron metabolism through its link to ROS production and iron-sulfur cluster biosynthesis was associated with neuronal pathology [8]. CL externalization has been shown to induce mitophagy in primary cortical neurons and in response to PD-inducing toxins [5], hinting to the importance of this pathway in neuronal life and death. It is tempting to speculate that also Ca^2+^ signals that play a crucial role in neuronal function and survival might be a physiological mitophagy inducer, as e.g. FKBP8 contains a Ca^2+^/Calmodulin binding domain and its PPIase activity is sensitive to Ca^2+^ [3]. It is very probable that different mitophagy pathways amplify the regulatory potential of neurons to act to different physiological stimuli and cell type-specific requirements and that alterations in any of these pathways may result in neurodegeneration.
